# The Claims of Qualified Dentistry

**Published:** 1918-06-22

**Authors:** 


### THE CLAIMS OF QUALIFIED DENTISTRY.

STATISTICS recently communicated have shown that while the annual entry of medical students begins to recover from the upset of the war, and is reckoned in thousands, the net annual increase in the Dental Register is but sixteen. Of course, doctors are many times (in this country about thirteen times, to be exact) as numerous as qualified dentists. It has to be remembered, however, that whilst irregular medical practitioners—herbalists, quacksalvers, "healers," and the like—are largely excluded from employment, irregular dentists, those who hold no recognised diploma, or none at all, are not thus restrained, being, indeed, just as legally entitled to treat patients as are those who have been scientifically trained and tested. The dental profession is, in short, in a transition stage. But as it is the activities of its trained members which benefit the community far the most, these have an obvious claim to protection of their interests, even as against their greatly more numerous colleagues of lower standing. This Briarean war has not failed to touch the fortunes of dentists, as it has of all sorts and conditions of men. Indeed, the future supply of qualified dental surgeons, among whom women are a rarity, is endangered; and from instances privately reported to us, it seems likely that the already over-limited scope of qualified dentistry is being affected; that the poorer classes, at any rate, are being perforce consigned to the care of unqualified dental practitioners, upon whom the community generally is being encouraged to look as upon highly reliable persons. Here is one such instance. A town large enough to form a county borough had but one qualified dentist, pre-war. He possessed a medical qualification also, and on the outbreak of hostilities joined up to remain with the Forces ever since. The *locum tenens* who conducted his practice, being of military age, has now been warned that he will be liable for service. So that unless the advisory dental committee, under the highly efficient guidance of Mr. Dolamore, can secure the services of an over-age qualified man who is at liberty in these busy times, it looks as though a large town would be left as regards dental needs in the hands of the relatively less competent, while the business of a man at the Front would be destroyed. As to the gain to set against these things, that is nothing to speak of. It is said that no more dentists are to be enlisted as private soldiers, at least only for a very short period, and that dental commissions are to be awarded them almost immediately upon their donning uniforms. Their work will, of course, then be to attend to the dental needs of soldiers. This, however, the *locum tenens* had already been doing to the extent of a great deal of his time, as there were one or two camps near his practice.

Again, an instance has occurred where a commercial dental undertaking conducted by a salaried unqualified dentist under lay control is still allowed petrol to run a very large showy-looking car which generally conveys only the driver, the manager aforesaid, but in which he has lately picked up patients and driven them to his premises. Yet, in the same area, the newly appointed qualified school dentist and nurse, who have to reach remote country schools, have been told by their employers, the County Council, that it would be impossible to get a petrol licence for even a motor bicycle. This, at least, is a state of things which calls for immediate action, and the authorities concerned might well cut down the petrol allowance in the first case by half, for these are no times in which to suffer advertisement of such a nature to go on, let alone the favouring of inferior treatment on commercial lines over the scientific dental care of the children of a large district.

We have said enough, perhaps, to show that the proper evolution of the dental profession to a form and stage already, happily, reached by the medical one, is being somewhat endangered by current conditions; and, moreover, very largely, needlessly endangered. This is no academic matter merely. The relation of teeth to health is so close that hygienic claims of the highest importance are involved. We hope that all concerned will do all in their power to conserve and improve the position of qualified dentistry.

